# Inhibitors of glutamate release from breast cancer cells; new targets for cancer-induced bone-pain

**DOI:** 10.1038/srep08380

**Published:** 2015-02-11

**Authors:** Jennifer Fazzari, Hanxin Lin, Cecilia Murphy, Robert Ungard, Gurmit Singh

**Affiliations:** 1 Michael G. DeGroote Institute for Pain Research and Care, Department of Pathology and Molecular Medicine, McMaster University, Hamilton, Ontario Canada; 2 Centre for Microbial Chemical Biology, McMaster University, Hamilton, Ontario Canada

## Abstract

Glutamate is an important signaling molecule in a wide variety of tissues. Aberrant glutamatergic signaling disrupts normal tissue homeostasis and induces several disruptive pathological conditions including pain. Breast cancer cells secrete high levels of glutamate and often metastasize to bone. Exogenous glutamate can disrupt normal bone turnover and may be responsible for cancer-induced bone pain (CIBP). CIBP is a significant co-morbidity that affects quality of life for many advanced-stage breast cancer patients. Current treatment options are commonly accompanied by serious side-effects that negatively impact patient care. Identifying small molecule inhibitors of glutamate release from aggressive breast cancer cells advances a novel, mechanistic approach to targeting CIBP that could advance treatment for several pathological conditions. Using high-throughput screening, we investigated the ability of approximately 30,000 compounds from the Canadian Compound Collection to reduce glutamate release from MDA-MB-231 breast cancer cells. This line is known to secrete high levels of glutamate and has been demonstrated to induce CIBP by this mechanism. Positive chemical hits were based on the potency of each molecule relative to a known pharmacological inhibitor of glutamate release, sulfasalazine. Efficacy was confirmed and drug-like molecules were identified as potent inhibitors of glutamate secretion from MDA-MB-231, MCF-7 and Mat-Ly-Lu cells.

Bone metastasis is a common characteristic of advanced, highly aggressive breast cancer^1^. A high proportion of breast cancer patients presenting with bone metastases experience significant co-morbidities such as bone fracture and hypercalcemia^2^,^3^. The most prominent, however, is the manifestation of severe, intractable cancer-induced bone pain (CIBP)^4^. This unique chronic pain state can significantly compromise patient quality of life and functional status. Furthermore, therapeutic strategies for severe cancer pain are often constrained by dose-limiting side effects and acquired treatment resistance. The satisfactory management of chronic pain is essential to successful palliative care in cancer patients. In patients with tumours, 15–75% present with significant chronic pain. While pain management is increasingly a priority in cancer care, the cancer-induced pain state is poorly characterized and treatment outcomes can frequently exacerbate the poor quality of life experienced by most patients^5^. As CIBP has been demonstrated to be a unique pain state distinct from other chronic pain conditions^6^, there is the potential and the need to develop unique treatments for CIBP. Investigating and targeting the factors that initiate CIBP may allow for the development of effective therapeutics with minimal side effects. Investigating the effects of tumour-secreted factors on the host microenvironment, such as the bone, will provide insights into the physiological mechanisms underlying CIBP. In turn, this will aid in the development of novel pharmacological strategies for targeted pain interventions.

Glutamate is both an ubiquitous cell-signaling molecule in many tissues and a well-characterized excitatory neurotransmitter in the central nervous system (CNS), where it is involved in nociception and pain sensitization^7^,^8^. Both metabotropic and ionotropic glutamate receptors are involved in pain hypersensitivity^9^, and glutamate secretion is associated with peripheral tissue injury and inflammation^10^,^11^. Glutamate is also implicated peripherally in a variety of non-malignant painful states including polymyalgia^12^, arthritis^13^,^14^ and other inflammatory disorders^10^,^15^. Therefore, glutamate plays a key role in both central and peripheral propagation of pain including the development of features of chronic pain and hypersensitivity. In addition to its role in the CNS, glutamate is also an important metabolic component and signaling molecule in the periphery^16^,^17^. Among the spleen, pancreas, lung, heart, liver and other organs of the digestive and reproductive system, bone is also sensitive to glutamatergic signaling^18^,^19^. In the restricted environment of the bone, glutamate acts in an autocrine and paracrine manner, coordinating intra- and intercellular communication between prominent cells of the bone such as osteoblasts and osteoclasts. Signaling between these cells coordinates bone deposition and resorption in a glutamate-dependent manner^19^,^20^,^21^.

Intracellular glutamate is primarily a product of glutamine metabolism in cancer cells with a proportion of this glutamate pool destined for secretion^22^,^23^,^24^. In cancer cells, amplified secretion of glutamate, as well as other aspects of dysregulated glutamatergic signaling, have been shown to correlate with a malignant phenotype^25^,^26^,^27^. For example, exogenous glutamate secretion from glioma cells in the CNS allows tumour expansion and metastasis through excitotoxic cell death of proximal neurons and glial cells^28^. In the periphery, cancer cell lines including breast and prostate cancers associated with bone metastases also exhibit increased secretion of glutamate that contributes to the disruption of normal bone homeostasis and CIBP^21^.

Increased glutamine consumption is a hallmark of many neoplasms and cancer cells. Many aggressive breast cancer cell lines have been observed to be glutamine auxotrophs^29^. Glutamine is the major energy source for many tumours, as it is able to meet the bioenergetic demands of cancer cells while providing macromolecular intermediates that are required for rapid growth and proliferation^30^. Glutamine metabolism is initiated by the glutaminase-mediated conversion of L-glutamine to L-glutamate. With further processing by glutamate dehydrogenase, the resulting product, α-ketoglutarate, can directly enter the TCA cycle. Furthermore, glutamine metabolism provides molecular precursors for glutathione synthesis which maintain redox equilibrium in rapidly proliferating cancer cells^31^,^32^. In malignancies, the demand for glutamine rapidly surmounts its endogenous supply, exceeding that needed for biosynthetic processing alone^33^. Generally classified as a non-essential amino acid, an exogenous glutamine supply becomes essential for cancer cell metabolism and survival.

Glutamate signaling involves several classes of receptors. In transformed cells, metabotropic glutamate receptors have been shown to confer oncogenic potential^34^,^35^. Such G-protein coupled receptors with oncogenic activity are associated with increased local levels of their ligand. The production of a ligand such as glutamate, by either the tumour itself or surrounding tissue promotes ectopic expression and continued activation of its receptors^36^,^37^. The growth of several types of tumours such as glioma^26^,^38^, breast cancer^27^ and melanoma^25^,^39^ have been attenuated by inhibiting glutamatergic signaling in xenografts and cultured cell lines.

A variety of mechanisms may affect the secretion of glutamate from cancer cells. In addition to pathways that produce an intracellular source of glutamate, mechanisms that transport this amino acid across the plasma membrane should be considered as targets for pharmacological inhibition. Notably, breast and prostate cancer cells secrete high concentrations of glutamate through the activity of the cystine/glutamate antiporter, system x_c_^−^ 24,38. Survival of these tumours, amongst others, is dependent on this system, where its inhibition affects cell growth and viability^40^,^29^. System x_C_^−^ is a Na^+^-independent, anionic amino acid transporter^41^. It is composed of heavy and light chain subunits, 4F2hc and xCT, respectively^42^. A ubiquitous glycoprotein, 4F2hc facilitates the transport of the light chain to the plasma membrane^43^. The light chain, xCT, is an integral membrane protein with twelve transmembrane domains. It is this subunit that confers specificity to this transport system, facilitating the 1:1 exchange of the anionic form of cystine for L-glutamate^44^. MDA-MB-231 triple-negative breast cancer cells express several glutamate receptors and transporters driving both glutamate secretion and uptake^38^. It has been shown that glutamate secretion from these cells is limited by an inhibitor of system x_c_^−^, sulfasalazine (SSZ)^45^. However, inhibition of the vesicular glutamate transporter (VGLUT-1) does not affect glutamate release^38^. This suggests that a large proportion of glutamate is secreted through system x_c_^−^. In addition, we have previously shown that in a mouse model of CIBP, treatment with SSZ attenuated pain behaviours in mice harbouring intrafemoral MDA-MB-231 xenografts^46^. Furthermore, the excitotoxic levels of glutamate release from glioma is also attributed to the activity of system x_C_^−^47. System x_C_^−^ inhibition has also demonstrated advantageous results in several other cancer-associated pathologies. These include inducing a reduction in epileptic seizures associated with glioma^48^, decreasing cellular resistance to chemotherapy^49^, and increasing cell susceptibility to oxidative stress, leading to greater cancer cell death^50^. Although a widely utilized drug for ulcerative colitis and rheumatoid arthritis, SSZ is not an immediately viable therapeutic option for system x_C_^−^ inhibition due to its limited bioavailability when administered orally. The inhibitory action of SSZ on system x_C_^−^ is dependent on the whole molecule not its colonic metabolites, sulfapyridine and 5-aminosalicylic acid^40^,^45^. It is therefore of considerable interest to identify other compounds that act on a target such as system x_C_^−^ in order to treat CIBP. Inhibiting the release of glutamate from the cancer cells themselves is a novel, mechanistic strategy to eliminate the causative agent of several pathological conditions caused by metastatic cancer, including severe CIBP.

## Results

### High-throughput screening for the inhibition of glutamate release from MDA-MB-231 cells

A live cell-based screen was used for primary screening to achieve physiologically relevant results and thus foster the selection of higher-quality candidate compounds. In the Amplex Red assay, L-glutamate present in cell culture media is measured indirectly based on the level of H_2_O_2_ produced from the oxidation of L-glutamate by L-glutamate oxidase (producing α-ketoglutarate, NH_3_, and H_2_O_2_). Peroxide production is then quantified through the generation of a fluorescent product, resorufin by a horseradish peroxidase (HRP)-catalyzed reaction with the Amplex Red reagent. Initially, the Z′ factor was calculated to identify the statistical window to assess the effectiveness of the Amplex Red assay for extracellular glutamate in high-throughput screening (HTS). This window exists at least 3 standard deviations below the extracellular glutamate levels of the high control, DMSO, and 3 standard deviations above the low control, SSZ after a 48 hour incubation. The Z′ factor calculated for production of the fluorescent product of the assay, resorufin, was consistently higher than 0.6. With the maximum Z′ factor being 1, this assay was determined to be suitable for HTS.

**Figure 1:**
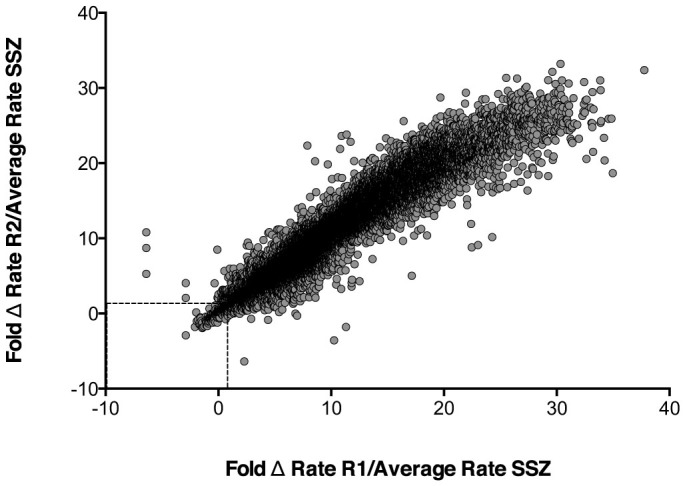
High-throughput screening of 29,586 compounds for inhibitors of glutamate release by MDA-MB-231 cells. The level of glutamate in the cell medium was measured by Amplex Red reagent 48 hours post inoculation. Fluorescence was read every 90 seconds for 15 minutes. The rate change of fluorescent signal versus time was used as indicator of inhibitory potency. The result was expressed as the ratio of rate change between a testing compound (10 μM) and the positive control, SSZ (200 μM). The X and Y-axis represent the ratio of rate change of replicate 1 (R1) and replicate 2 (R2). Among 500 positive hits, 320 (enclosed in box) showed similar to more potent glutamate release inhibition potency as SSZ and were selected for secondary screening.

**Figure 2:**
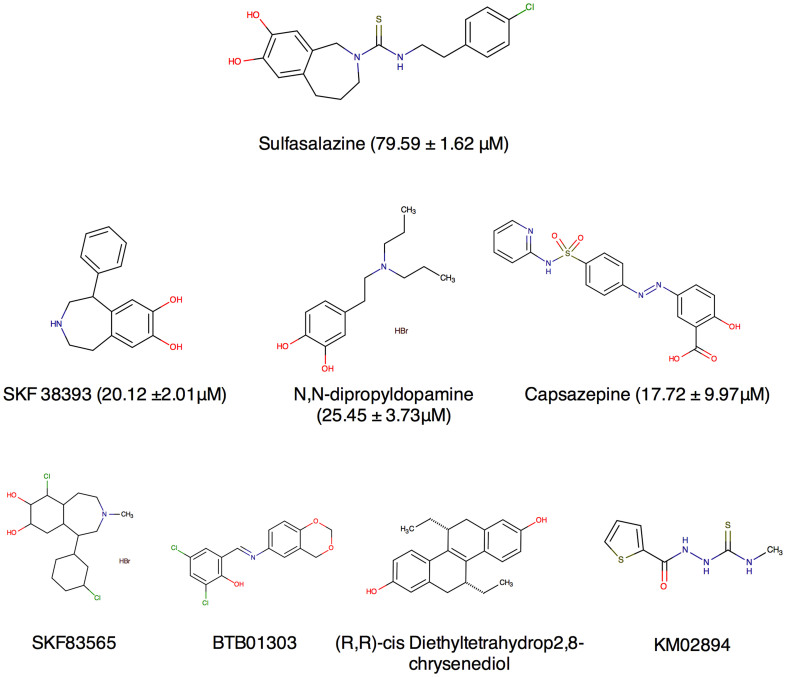
Chemical structure of 8 compounds showing potent inhibition of glutamate release after secondary screening. Positive hits selected from HTS screening as inhibitors of glutamate release from MDA-MB-231 cells. The remaining compounds were those that were found under HTS conditions to inhibit glutamate release but were not tested in follow-up studies.

**Figure 3:**
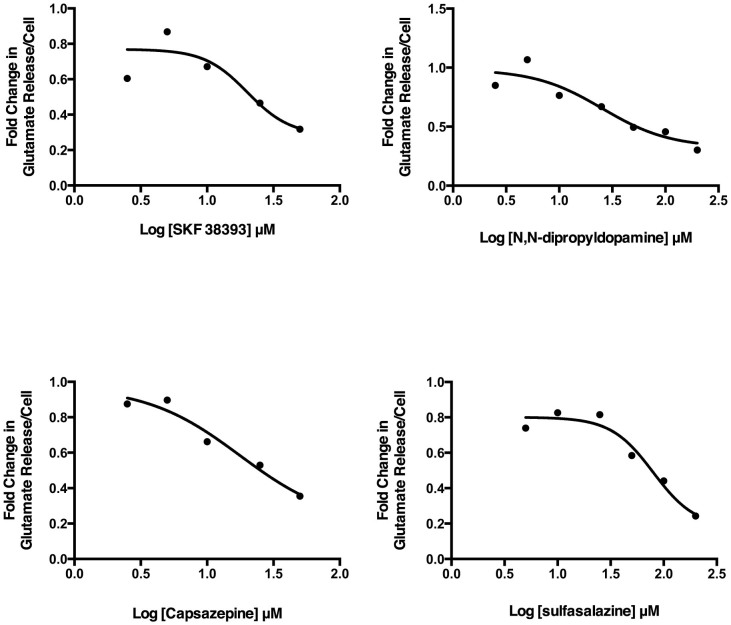
IC_50_ curves for capsazepine, NNDP, SKF38393 and sulfasalazine. The IC_50_ was calculated by normalizing to the viable cell numbers. The IC_50_ values are as follows: capsazepine = 17.72, SKF38393 = 20.12, NNDP = 25.45 SSZ = 79.59 μM.

**Figure 4:**
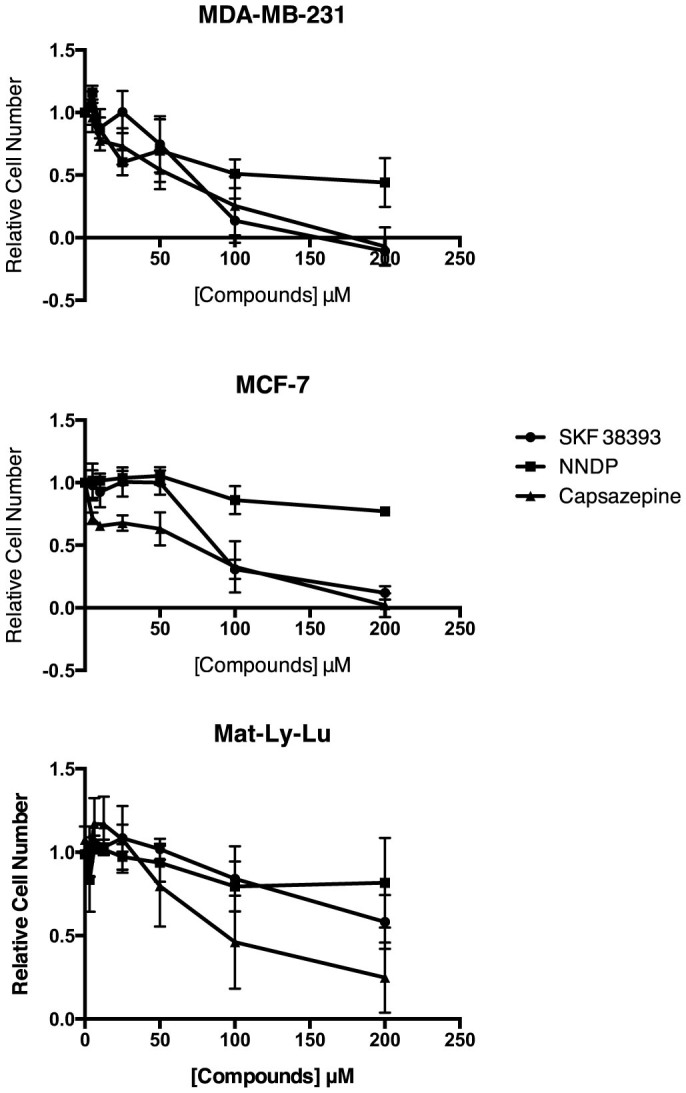
Cytotoxicity of SKF38393, NNDP and capsazepine in MDA-MB-231, MCF-7 and Mat-Ly-Lu cells. The cell number was quantified by crystal violet staining 48 hours post-incubation. Data are represented as the mean of n = 3 experiments ± the standard error of the mean.

Fluorescence was measured 10 times at 90 second intervals for each plate. The rate change of relative fluorescent units versus time was calculated and used as indicator of inhibitor potency. The results were expressed as a ratio of the rate change between a testing compound and the positive control, SSZ (Fig. 1). A ratio of 1.0 means that the test compound, at a 10 μM screening concentration, has the same potency of inhibition as 200 μM SSZ. Approximately 500 positive hits were identified from the primary screening. Among these compounds, 110 were 0.2-fold (i.e. 5 times) more potent than SSZ, 127 were less than 0.4-fold, 292 were less than 1-fold, and 320 were less than 1.1-fold. These 320 compounds were selected for secondary screening. During secondary screening, cell viability was assessed visually following treatment with the compounds selected from the primary screen. A potent cytotoxic compound would not qualify as a viable therapeutic candidate, as it most likely would have targets outside the tumour in healthy tissue when administered *in vivo*. Eliminating these compounds with potent cytotoxicity narrowed the range of positive hits before progressing to secondary screening. Ultimately, 7 compounds were identified as viable potent inhibitors of glutamate release with low to moderate cytotoxicity. These compounds were (R,R)-cis-Diethltetrahydro-2,8-chrysendiol, (+/−)-SKF38393 hydrochloride, N,N-dipropyldopamine hydrobromide (NNDP), capsazepine, SKF83565 hydrobromide, KM02894 and BTB01303 (Fig. 2). Among them, SKF38393, SKF83565 and NNDP are well-characterized dopamine receptor agonists, while capsazepine is a vanilloid receptor antagonist. Interestingly, these four compounds share a substituted benzazepine functional group. Substituted benzazepine derivatives (1-phenyl-1H-3-benzazepines) have been shown to have specificity for the dopamine D1 receptor^51^. Due to supplier availability, SKF83565 and KM02894 were not available for subsequent testing. (R,R)-cis-Diethltetrahydro-2,8-chrysendiol and BTB01303 were eliminated due to structurally predicted auto-fluorescence that may interfere with the glutamate release assay. As a result, SKF38393, NNDP and capsazepine were selected for follow-up testing to assess their cytotoxicity and inhibitory effect on glutamate release in a 96-well plate format. Ultimately, capsazepine, SKF38393 and NNDP showed a dose dependent inhibition of glutamate release (Fig. 3) and low to moderate cytotoxicity (Fig. 4).

### IC_50_ Values for SKF 38393, N,N-dipropyldopamine and Capsazepine

In the Amplex Red reaction, glutamate is initially converted to α-ketoglutarate by glutamate oxidase, which produces H_2_O_2_. This initiates the HRP-catalyzed reaction with the Amplex Red reagent to generate the fluorescent product, resorufin, which is quantified to indirectly measure glutamate. Therefore, the final fluorescent readout is potentially affected by H_2_O_2_ released by the cells when testing a range of concentrations. Our data showed that H_2_O_2_ production was not a confounding factor except at high doses of SKF38393 and capsazepine (≥100 μM; data not shown). These concentrations were eliminated from further testing. SKF38393, NNDP and capsazepine had IC_50_ values lower than that of SSZ, suggesting greater potency than the positive control. After normalizing to viable cell number quantified 48 hours post inoculation, the IC_50_ of capsazepine, SKF38393, NNDP and SSZ was calculated as 17.72, 20.12, 25.45 and 79.59 μM, respectively (Fig. 4; Table 1). Therefore, capsazepine, SKF38393 and NNDP are more potent inhibitors of glutamate release from cancer cells than SSZ. This trend was also reflected in the human MCF-7 breast adenocarcinoma and the rat Mat-Ly-Lu prostate cancer line with the exception of capsazepine that did not show effective glutamate inhibition in these lines (Table 1).

## Discussion

The objective of this study was to identify small molecule inhibitors of glutamate secretion from human cancer cells. HTS of small molecules is an important stage of drug discovery. HTS allows for the identification of new agents that target glutamate release from aggressive, metastatic cancer cell lines that we have previously shown to release glutamate and one of which was used to induce a cancer-induced bone pain state^46^. This investigation represents a novel approach to treating cancer pain and is a stepping-stone in developing new, targeted therapeutic strategies for this unique chronic pain state. Current pain interventions offer pain management strategies but are generally unable to address the unique etiology of cancer-pain. Most commonly consulted is the World Health Organization's Pain Ladder that suggests the gradual progression from non-steroidal anti-inflammatories to drugs that have increasing analgesia with strong opioids suggested for the most intense forms of cancer pain. Despite this, such pharmacological interventions still leave a proportion of patients with inadequate pain control^52^. A comprehensive review of a wide array of cancer-related bone pain interventions is reviewed by Mercadante, 1997 and includes, in addition to analgesics, the use and limitations of radiotherapy to ablate local bone pain due to tumour mass, chemo/hormonal therapy which correlates tumour growth inhibition to the alleviation of pain, bisphosphonates preventing bone lesions associated with osteoclast activity amongst other interventions including psychiatric and invasive approaches^4^. CIBP is generally composed of a chronic, dull pain compounded by both incidental and spontaneous episodes of severe pain. The intermittent nature of this pain state often responds poorly to current pharmacological pain interventions where the degree of analgesia cannot be achieved without producing unacceptable side-effects in the patient^4^,^52^,^53^.

As a means of identifying potential mechanisms that contribute to excess glutamate release from cancer cells, the small molecules selected from our HTS implicate several novel molecular targets. Due to the high metabolic activity of cancer cells, the production of antioxidants must be upregulated to effectively maintain redox equilibrium. Glutamate release from cancer cells is thought to be a byproduct of a protective mechanism against oxidative stress. Synthesis of glutathione (GSH), the predominant cellular antioxidant, relies on the acquisition of cysteine as the rate-limiting step. Cysteine is acquired extracelullarly in its oxidized form, cystine, through the action of the cystine/glutamate antiporter system x_C_^−^. This transport activity, which is upregulated in cancer cells, necessitates the release of glutamate and is responsible for the majority of glutamate release in several cancer cell lines.

The inhibition of intracellular glutaminase is another potential mechanism that would affect the concentration of intracellular glutamate available for secretion. Collins et al.^23^ have shown that approximately 30% of secreted glutamate is derived from imported glutamine by way of glutaminase activity. Should any of our molecules disrupt glutaminase activity, the proportion of glutamate available for export would decrease. Glutamate secretion in cancer cells is affected by the extracellular concentration of cystine, and intracellular glutaminase activity does not correlate with glutamine consumption in breast cancer cells^23^. This suggests that glutaminase activity alone cannot account for changes in glutamate secretion. The majority of exported glutamate is however, coupled to the import of cystine^23^, again supporting a role for system x_C_^−^ in the secretion of a large proportion of released glutamate. This is consistent with the observations of Bannai and Ishii in fibroblasts^54^. Furthermore, we have shown that system x_C_^−^ activity is also associated with CIBP, where a known inhibitor of this antiporter, SSZ, reduces pain behaviours linked to the growth of MDA-MB-231 tumours in the distal femur^46^. While the compounds identified in our screen may inhibit glutamate release by a variety of mechanisms, system x_C_^−^ is likely a major target.

The known functions of SKF38393 and N,N-dipropyldopamine as dopamine receptor agonists, offers additional mechanisms that warrant further investigation into the role of the dopamine signaling pathway in malignant cells. The D1 dopamine receptor is linked to downstream activation of adenylyl cyclase and cAMP production^55^. Agonist versus antagonist activity of 1-phenyl-1H-3-benzazepines is dependent on the substituent occupying position 7 of the benzazepine molecule^56^. All molecules identified in our screening that contain the benzazepine group show agonistic properties by this method. The D1 receptor is expressed by breast cancer cells^57^, and dopamine itself is an effective adjuvant to increase the efficacy of anticancer agents^58^. Furthermore, there is evidence linking dopamine agonists to the functional reversal of the GLT-1 transporter^59^. The GLT-1 transporter has been shown by our laboratory to be present at the mRNA level in MDA-MB-231 cells and may therefore contribute to glutamate secretion in these cells^38^.

In addition to dopamine signaling, the Transient Receptor Potential cation channel 1 (TRPV1) may play a role in glutamate secretion. Also known as the type 1 vanilloid receptor, this ion channel is well characterized in pain pathways, with the excitotoxin capsaicin being a common agonist. One of the compounds identified in our screen, capsazepine, is a synthetic analog of capsaicin, that acts as a TRPV1 antagonist^60^. With well-characterized neurological effects, capsazepine has also been shown to mediate anticancer activity through a reactive oxygen species (ROS)-mediated JNK signaling mechanism^61^. TRPV1 receptors are present on tumour cells, however, the mechanism of action of vanilloids on these cells was not through the conventional calcium signaling associated with TRVP1 activation^62^. With potential mechanisms outlined, the mode of action of all the glutamate release-inhibiting compounds discovered are currently under investigation.

## Conclusion

Glutamate release is involved in several painful conditions and the cell-based HTS described in the current investigation has discovered several molecules that inhibit glutamate release. Previous studies by our lab have identified system x_C_^−^ as a major mechanism of glutamate release from cancer cells^38^. Several compounds identified in the screen suggest that, in addition to system x_C_^−^, other pathways and receptors may be at play. Our data suggest that dopamine signaling and TRPV1 activity may modulate glutamate release in MDA-MB-231 cells. We aim to further investigate the mode of action of these compounds, as target validation may contribute to the development of novel therapeutics for the treatment of several cancer-associated pathologies including glutamate-mediated CIBP. Our study represents a unique opportunity to study the mechanisms responsible for glutamate release from several metastatic cancer cell lines in an effort to use these compounds as tools to study glutamate signaling and how its inhibition translates to analgesia in a cancer-induced pain model. To pharmacologically inhibit pain propagation and hypersensitivity without affecting systemic signaling is a unique strategy for the development of novel therapeutics that address the underlying mechanism causing the pain rather than the development of those that merely mask pain intensity. Pursuing these efforts is the goal of future investigation into the means by which these molecules inhibit glutamate release from cancer cells.

## Methods

### Cell Culture

MDA-MB-231 human breast adenocarcinoma cells were maintained in high glucose DMEM (Life Technologies, Carlsbad, CA) supplemented with 10% fetal bovine serum (FBS) and 1% antibiotic/antimycotic (Life Technologies). Cells undergoing screening were maintained in DMEM supplemented with 10% dialyzed FBS (dFBS) and 1% antibiotic/antimycotic (A/A; Life Technologies). All cells were incubated at 37°C and 5% CO_2_.

### Assay Optimization for High-Throughput Screening

Calculation of Z′ factor is used to assess the size of the screening window, which statistically outlines the region in which positive hits will be selected. The fluorescent signal produced from the Amplex Red reagent (Life Technologies) was found to be significantly above background signal. Measurements were taken over 13 time points within 83 minutes and the Z′ factor was calculated using the following equation (Equation 1):
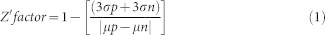
σ = standard deviation of positive (p) and negative (n) controls

μ = mean of positive (p) and negative (n) controls

### Cell-Based High-Throughput Screening for Molecules Inhibiting Glutamate Release

MDA-MB-231 cells were grown in T-75 flasks containing normal growth media as outlined above. Cells were harvested at 70–90% confluency with 0.5% trypsin/EDTA, counted by haemocytometer, and dispensed at a density of 700 cells per well of a 384 well-plate in DMEM supplemented with 10% dFBS and 1% A/A. Immediately after seeding, compounds of the Canadian Compound Collection library were dispensed at a concentration of 10 μM. All seeding, treatments, and subsequent assays were automated using a BIOMEK FX liquid handler (Beckman Coulter, Brea, CA). All compound plates comprising the library contained technical replicates but did not contain positive and negative controls. Positive and negative controls were therefore prepared in parallel in one plate containing 200 μM sulfasalazine (positive control) and 1% DMSO (negative control) in screening media. Furthermore, in order to establish basal glutamate levels in the growth medium, a plate containing only media was dispensed and subjected to the same protocol for compound testing. All plates were then incubated for 48 hours at 37°C and 5% CO_2_.

### Screening Library

The Canadian Compound Collection HTS library consists of 29,586 compounds including synthetic small molecules, off-patent small molecules that are FDA approved, natural products, pharmacologically active small molecules and bioactives. Compound stocks were solubilized at 1 mM in 100% DMSO and validated as greater than 95% pure.

### Measurement of Glutamate Release

Extracellular glutamate levels were measured after 48 hours using the Amplex Red glutamic acid assay kit (Life Technologies). This assay is modified to increase sensitivity to low glutamate concentrations by removing the L-alanine and L-glutamate-pyruvate transaminase from the reaction^24^. After the 48-hour incubation, the Amplex Red reaction mixture was added to each well at a ratio of 1:2. Immediately following addition, the plate was measured fluorometrically by the EnVision 2102 multilabel reader (Perkin Elmer, Waltham, MA) in continuous assay mode. Readings were acquired every 90 seconds for a total of 15 minutes at an excitation wavelength of 530–560 nm and an emission wavelength of 590 nm. The fold difference between the negative (DMSO) and positive (SSZ) control was greatest at a time of 15 minutes post-addition of the Amplex Red reagent. The slope of relative fluorescent units versus time in seconds is used as indicator of inhibitory potency. The smaller the value for the slope, the less glutamate is in the medium.

### Data Analysis – Determining Hit-rate

The glutamate release values for each compound were plotted against their technical replicate and normalized to SSZ. The results were gated to highlight compounds that had a fold change in the inhibition of glutamate release ≤1.1 relative to SSZ. From initial screening, 320 compounds that met this criterion were considered for re-screening. Of these compounds, a significant proportion were eliminated due to observable cytotoxicity which was classified as a confounding factor contributing to false positives. These compounds were not pursued in follow-up experiments.

### Prioritization of Compound Hits and IC_50_ Determination

Re-screened compound hits were then tested in a 96-well plate format. Cells were seeded at 5,000–10,000 cells/well and compounds were added over a range of 0–200 μM in DMEM + 10% dFBS immediately after cell seeding. Cultures were incubated at 37°C for 48 hours from the time of plating and compound addition. Media was collected after incubation and diluted 1:10 for glutamate determination by Amplex Red assay. The Amplex Red reaction mix consists of 1X reaction buffer (0.1 M Tris-HCl, pH 7.5), 100 U/mL horseradish peroxidase, 5 U/mL L-glutamate oxidase, and 2.6 μg/mL of the Amplex Red reagent dissolved in DMSO. Media sample dilutions were added at a 1:1 ratio with the reaction mixture (25 μL of each). Fluorometric data was measured by rate as mentioned above in order to establish the change in fluorescent units over time. IC_50_ values were calculated from the non-linear regression of slope versus log concentration of each compound.

### Cell Number Quantification

Cell number was quantified by crystal violet staining in order to assess compound cytotoxicity. After media collection, each well was aspirated, rinsed with PBS and fixed in formalin for 30 minutes. Formalin was then removed and cultures were stained with a 0.25% crystal violet in 25% methanol for 15 minutes. Plates were then submerged in water and rinsed until the stain was completely removed. Once dry, crystal violet stain was solubilized with a solution of 0.05 M NaH_2_PO_4_ in 50% ethanol and read on a spectrophotometer (Biotek, Winooski, VA) at λ = 570 nm. Results are compared to standard growth curves generated for the cell line and cell number was interpreted from the equation of the standard curve. Calculated values were then used to normalize relative glutamate concentrations to cell number.

### Measurement of Assay Interference

To determine whether any molecules interfered with the Amplex Red reaction, standard glutamate concentrations were measured in the presence and absence of SKF38393, NNDP, capsazepine and SSZ in isolation. The standard L-glutamate concentrations ranged from 0–25 μM and dilutions were prepared fresh before each test. All drug dilutions were added to the glutamate standards at a ratio of 1:100 as used in follow-up testing to ensure each sample has identical volumes of DMSO. The total volume of glutamate, drug and reaction buffer was 25 μL.

### Production of hydrogen peroxide

Because the measurement of glutamate by Amplex Red is indirect it is the production of H_2_O_2_ that induces HRP-catalyzed conversion of the Amplex Red reagent (10-acetyl-3,7- dihydroxyphenoxazine) to its fluorescent product resorufin. To ensure drug treatment did not induce exogenous H_2_O_2_ production/release, media samples collected from treated cells were tested in the absence of L-glutamate oxidase to allow basal H_2_O_2_ levels to be quantified.

## Author Contributions

J.F. and H.L. contributed equally to the manuscript. H.L. completed optimization experiments before starting high-throughput screening and J.F. and H.L. conducted the primary and secondary screening of small molecules. C.M. designed the automated high-throughput protocol, prepared compound libraries and Z′ factor calculation, initial data analysis composing Figure 1 as well compound hit identification. J.F. conducted follow-up compound characterization including IC_50_ calculation and cytotoxicity. The manuscript was written by J.F. and R.U. contributed to manuscript writing preparation and editing. All authors read and approved the final manuscript.

## Figures and Tables

**Table 1:** IC_50_ values reported for each compound tested on MDA-MB-231 and MCF-7 human breast cancer lines and the Mat-Ly-Lu rat prostate cancer line. All cell lines were treated for 48 hours before collection of media for glutamate quantification using Amplex Red. Cell number determined by crystal violet staining of formalin-fixed cells

	SKF- 38393	N,N-DP	CPZ	SSZ
**MDA-MB-231**	20.12	25.45	17.72	79.59
**MCF-7**	19.58	26.3	N.D.	101
**Mat-Ly-Lu**	13.77	28.45	N.D.	29.69
